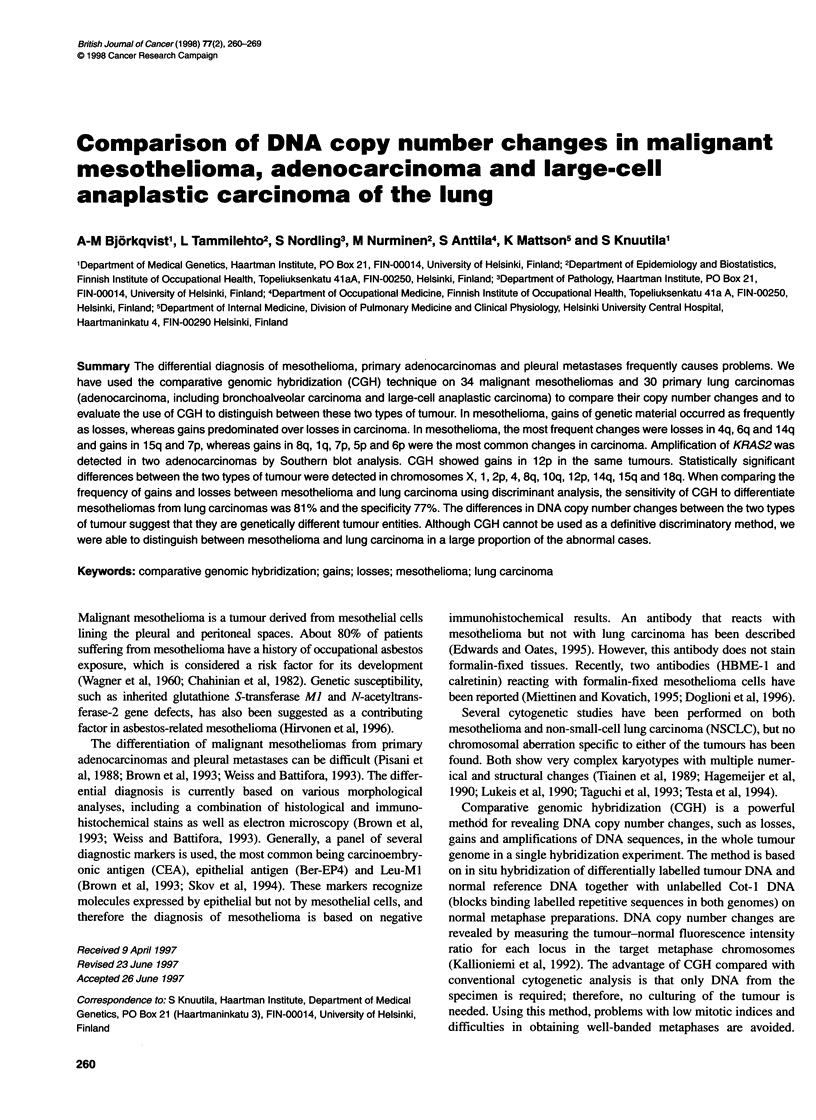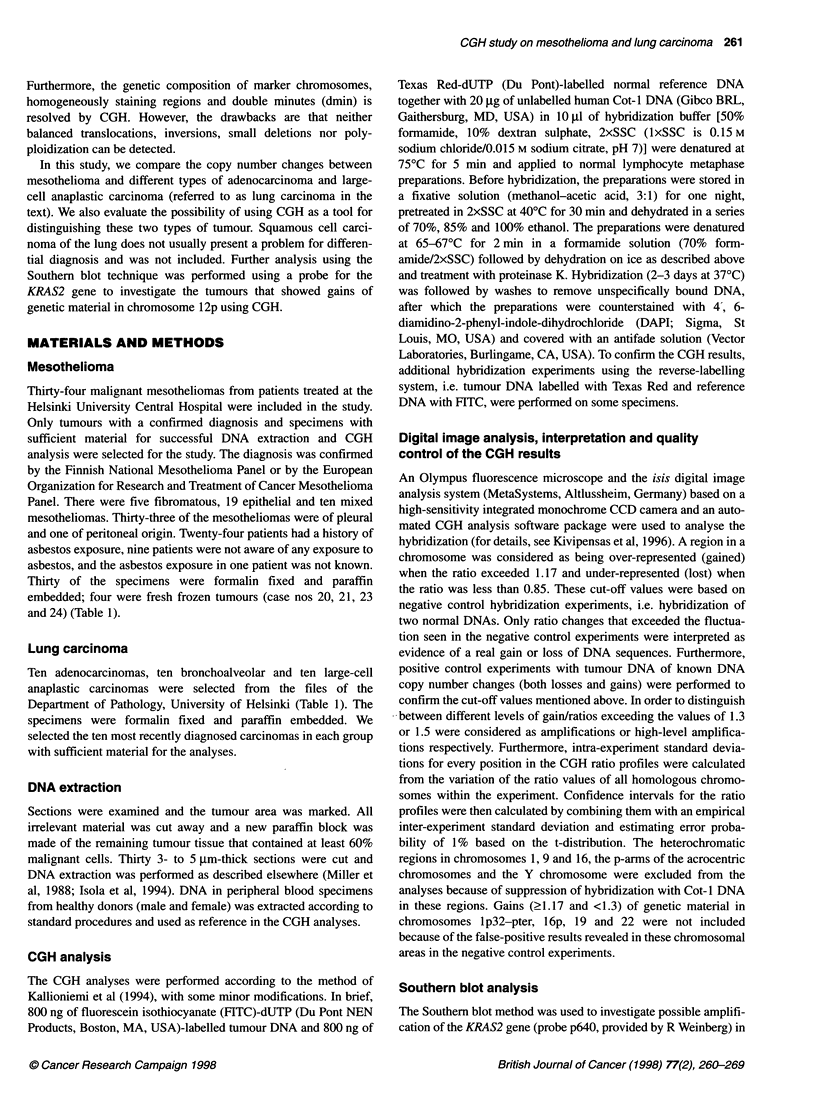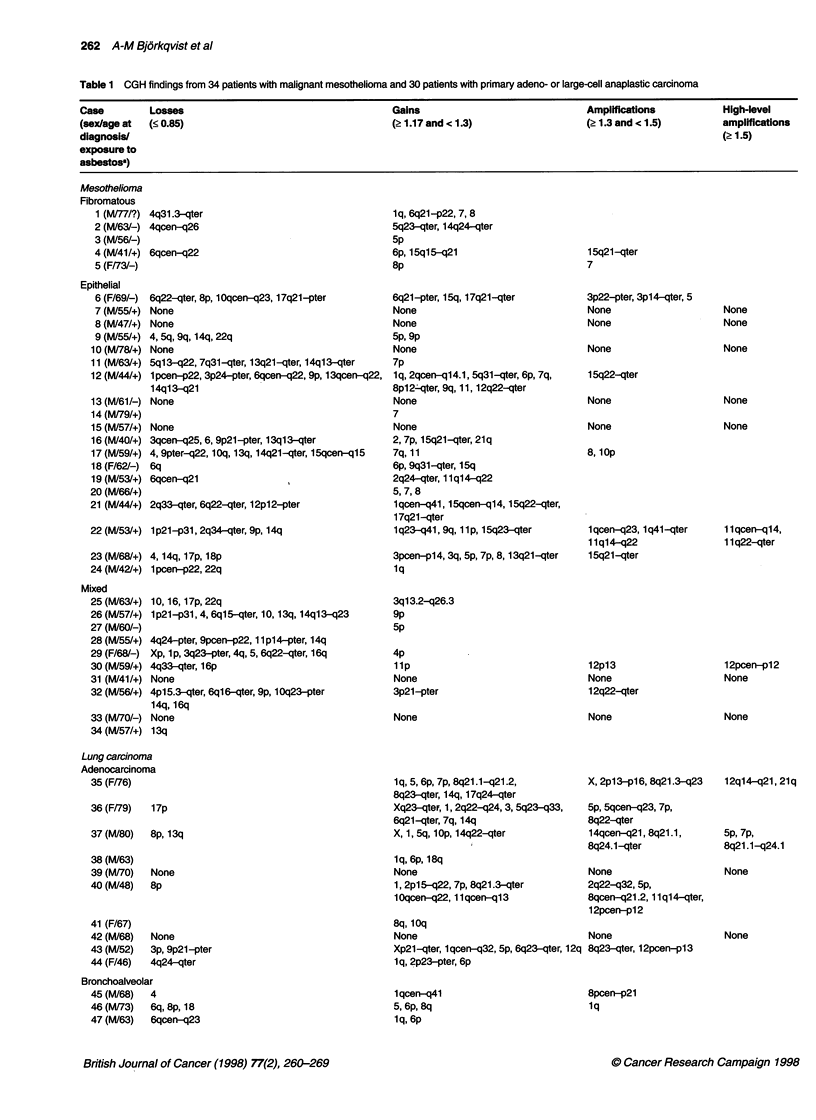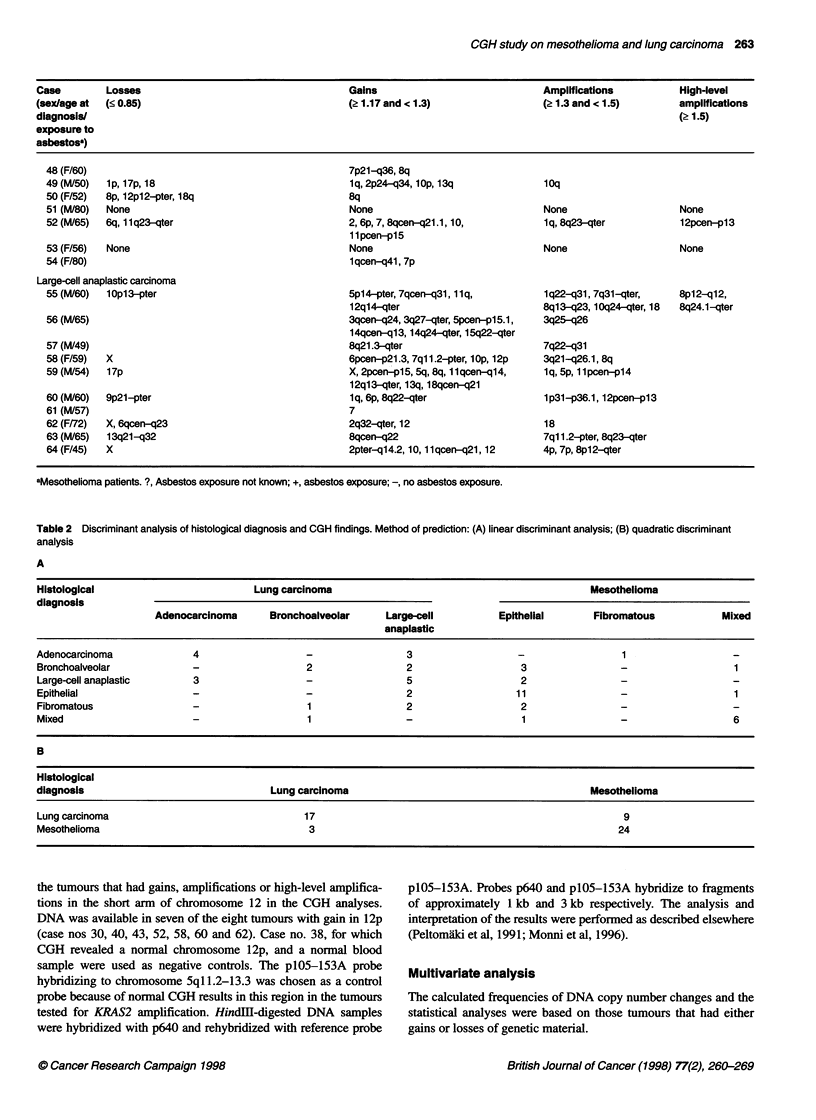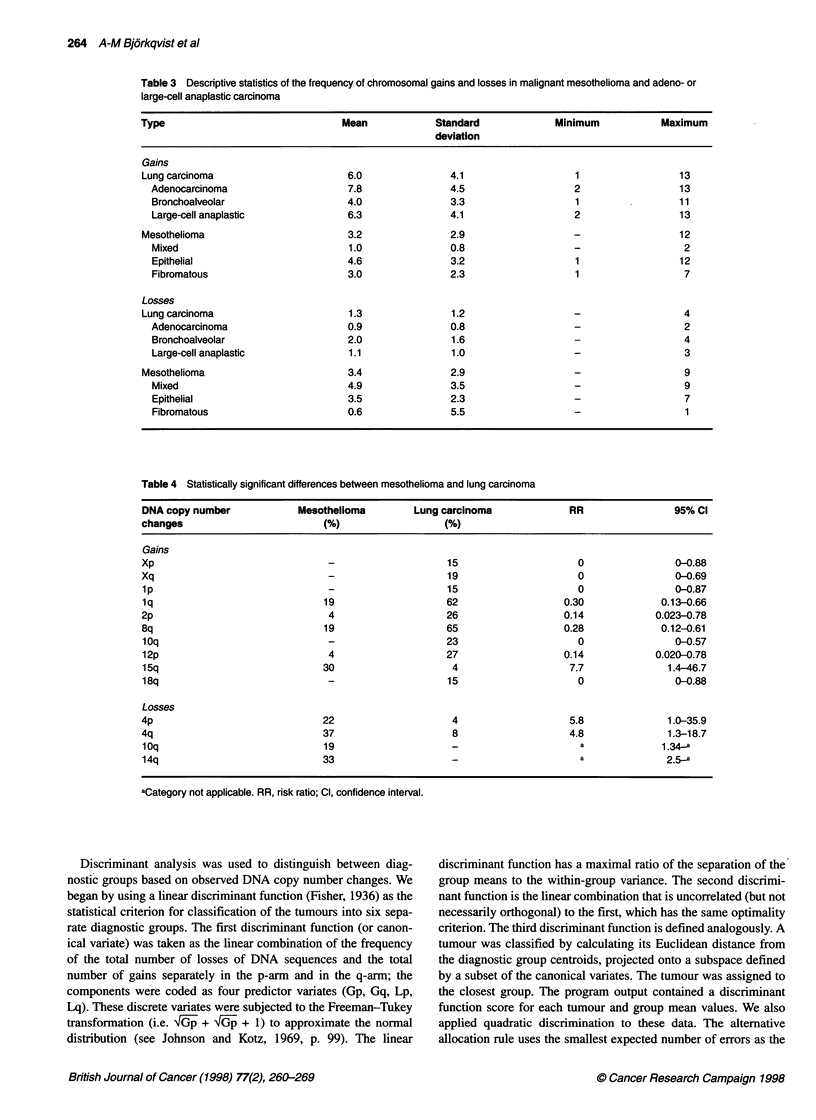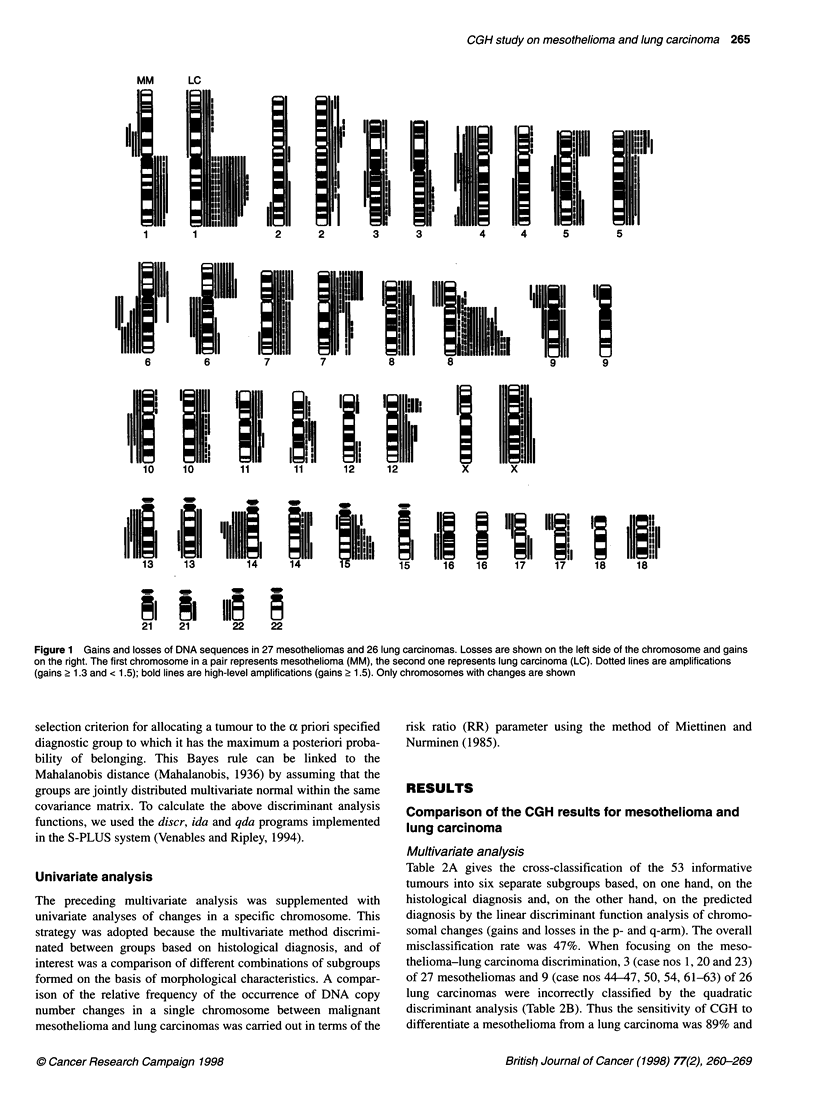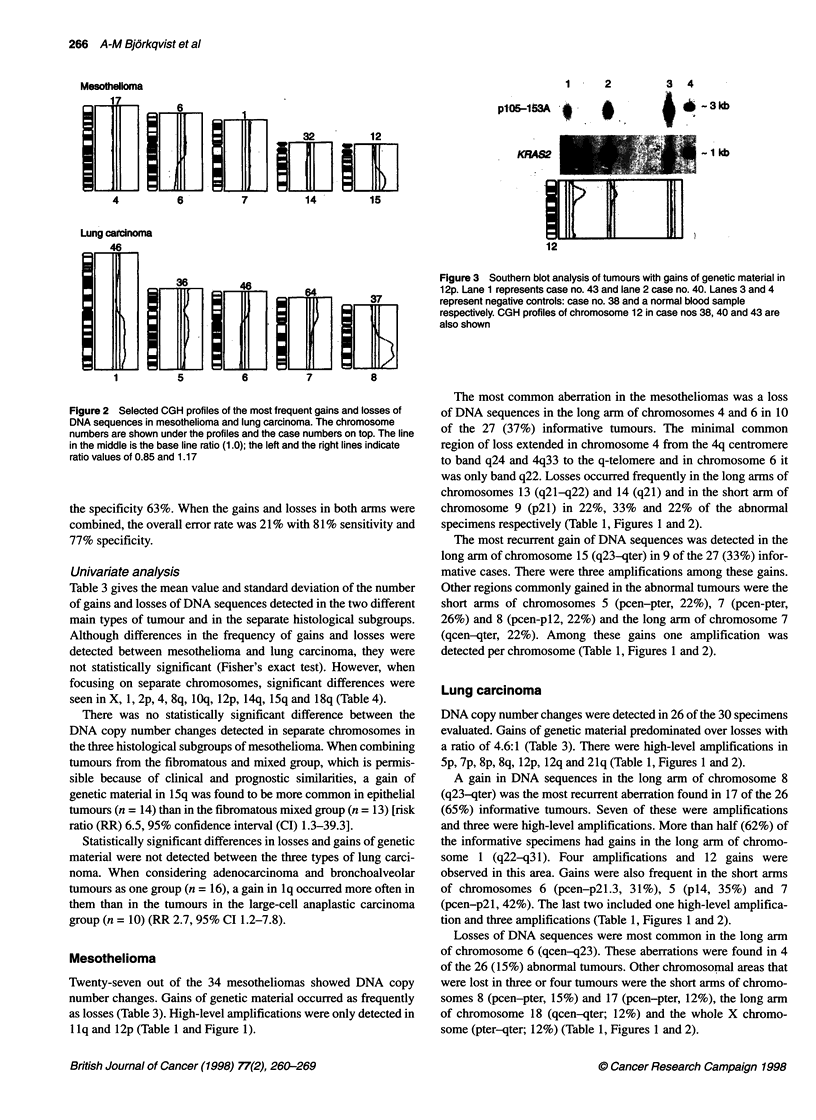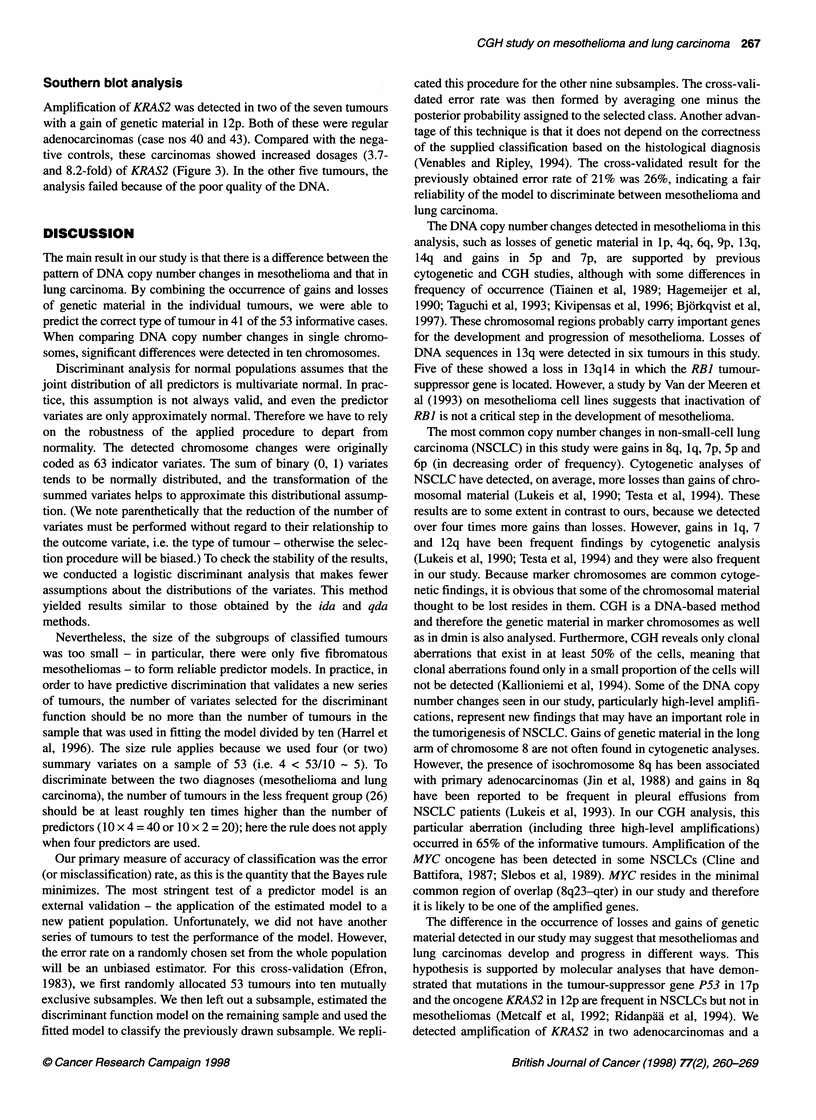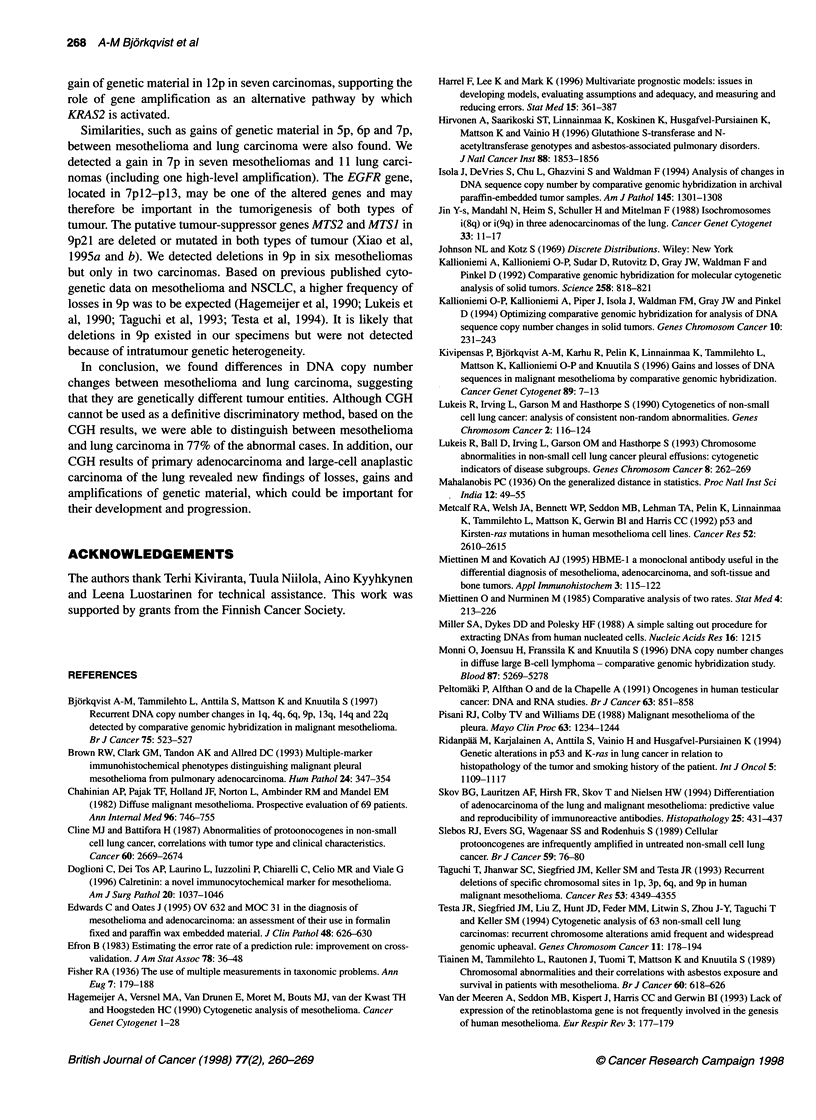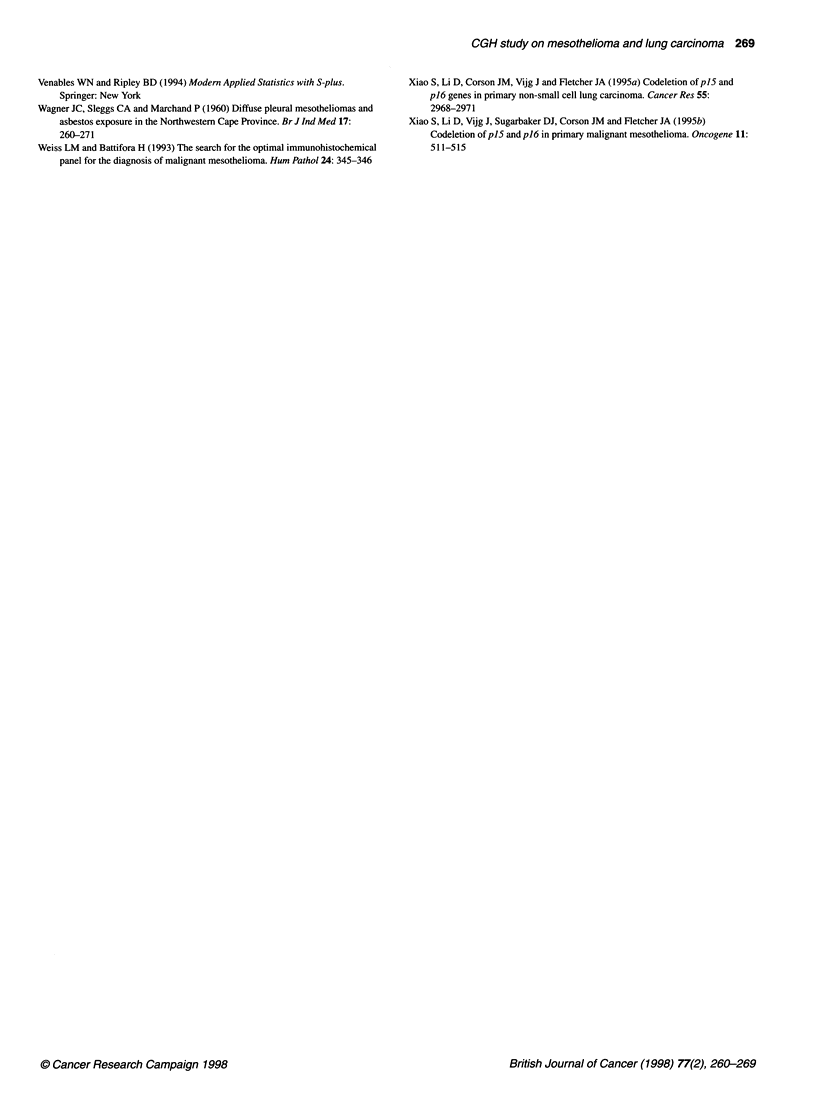# Comparison of DNA copy number changes in malignant mesothelioma, adenocarcinoma and large-cell anaplastic carcinoma of the lung.

**DOI:** 10.1038/bjc.1998.42

**Published:** 1998

**Authors:** A. M. BjÃ¶rkqvist, L. Tammilehto, S. Nordling, M. Nurminen, S. Anttila, K. Mattson, S. Knuutila

**Affiliations:** Department of Medical Genetics, Haartman Institute, University of Helsinki, Finland.

## Abstract

**Images:**


British Journal of Cancer (1998) 77(2), 260–269
© 1998 Cancer Research Campaign

### Comparison of DNA copy number changes in malignant mesothelioma, adenocarcinoma and large-cell anaplastic carcinoma of the lung

**A-M Björkqvist[1], L Tammilehto[2], S Nordling[3], M Nurminen[2], S Anttila[4], K Mattson[5] and S Knuutila[1]**

[1]Department of Medical Genetics, Haartman Institute, PO Box 21, FIN-00014, University of Helsinki, Finland; [2]Department of Epidemiology and Biostatistics, Finnish Institute of Occupational Health, Topeliuksenkatu 41aA, FIN-00250, Helsinki, Finland; [3]Department of Pathology, Haartman Institute, PO Box 21, FIN-00014, University of Helsinki, Finland; [4]Department of Occupational Medicine, Finnish Institute of Occupational Health, Topeliuksenkatu 41a A, FIN-00250, Helsinki, Finland; [5]Department of Internal Medicine, Division of Pulmonary Medicine and Clinical Physiology, Helsinki University Central Hospital, Haartmaninkatu 4, FIN-00290 Helsinki, Finland

**Summary** The differential diagnosis of mesothelioma, primary adenocarcinomas and pleural metastases frequently causes problems. We have used the comparative genomic hybridization (CGH) technique on 34 malignant mesotheliomas and 30 primary lung carcinomas (adenocarcinoma, including bronchoalveolar carcinoma and large-cell anaplastic carcinoma) to compare their copy number changes and to evaluate the use of CGH to distinguish between these two types of tumour. In mesothelioma, gains of genetic material occurred as frequently as losses, whereas gains predominated over losses in carcinoma. In mesothelioma, the most frequent changes were losses in 4q, 6q and 14q and gains in 15q and 7p, whereas gains in 8q, 1q, 7p, 5p and 6p were the most common changes in carcinoma. Amplification of *KRAS2* was detected in two adenocarcinomas by Southern blot analysis. CGH showed gains in 12p in the same tumours. Statistically significant differences between the two types of tumour were detected in chromosomes X, 1, 2p, 4, 8q, 10q, 12p, 14q, 15q and 18q. When comparing the frequency of gains and losses between mesothelioma and lung carcinoma using discriminant analysis, the sensitivity of CGH to differentiate mesotheliomas from lung carcinomas was 81% and the specificity 77%. The differences in DNA copy number changes between the two types of tumour suggest that they are genetically different tumour entities. Although CGH cannot be used as a definitive discriminatory method, we were able to distinguish between mesothelioma and lung carcinoma in a large proportion of the abnormal cases.

**Keywords:** comparative genomic hybridization; gains; losses; mesothelioma; lung carcinoma

Malignant mesothelioma is a tumour derived from mesothelial cells lining the pleural and peritoneal spaces. About 80% of patients suffering from mesothelioma have a history of occupational asbestos exposure, which is considered a risk factor for its development (Wagner et al, 1960; Chahinian et al, 1982). Genetic susceptibility, such as inherited glutathione *S*-transferase *M1* and *N*-acetyltransferase-2 gene defects, has also been suggested as a contributing factor in asbestos-related mesothelioma (Hirvonen et al, 1996).

The differentiation of malignant mesotheliomas from primary adenocarcinomas and pleural metastases can be difficult (Pisani et al, 1988; Brown et al, 1993; Weiss and Battifora, 1993). The differential diagnosis is currently based on various morphological analyses, including a combination of histological and immunohistochemical stains as well as electron microscopy (Brown et al, 1993; Weiss and Battifora, 1993). Generally, a panel of several diagnostic markers is used, the most common being carcinoembryonic antigen (CEA), epithelial antigen (Ber-EP4) and Leu-M1 (Brown et al, 1993; Skov et al, 1994). These markers recognize molecules expressed by epithelial but not by mesothelial cells, and therefore the diagnosis of mesothelioma is based on negative immunohistochemical results. An antibody that reacts with mesothelioma but not with lung carcinoma has been described (Edwards and Oates, 1995). However, this antibody does not stain formalin-fixed tissues. Recently, two antibodies (HBME-1 and calretinin) reacting with formalin-fixed mesothelioma cells have been reported (Miettinen and Kovatich, 1995; Doglioni et al, 1996).

Several cytogenetic studies have been performed on both mesothelioma and non-small-cell lung carcinoma (NSCLC), but no chromosomal aberration specific to either of the tumours has been found. Both show very complex karyotypes with multiple numerical and structural changes (Tiainen et al, 1989; Hagemeijer et al, 1990; Lukeis et al, 1990; Taguchi et al, 1993; Testa et al, 1994).

Comparative genomic hybridization (CGH) is a powerful method for revealing DNA copy number changes, such as losses, gains and amplifications of DNA sequences, in the whole tumour genome in a single hybridization experiment. The method is based on in situ hybridization of differentially labelled tumour DNA and normal reference DNA together with unlabelled Cot-1 DNA (blocks binding labelled repetitive sequences in both genomes) on normal metaphase preparations. DNA copy number changes are revealed by measuring the tumour–normal fluorescence intensity ratio for each locus in the target metaphase chromosomes (Kallioniemi et al, 1992). The advantage of CGH compared with conventional cytogenetic analysis is that only DNA from the specimen is required; therefore, no culturing of the tumour is needed. Using this method, problems with low mitotic indices and difficulties in obtaining well-banded metaphases are avoided.

*Received 9 April 1997*
*Revised 23 June 1997*
*Accepted 26 June 1997*

*Correspondence to:* S Knuutila, Haartman Institute, Department of Medical Genetics, PO Box 21 (Haartmaninkatu 3), FIN-00014, University of Helsinki, Finland

Furthermore, the genetic composition of marker chromosomes, homogeneously staining regions and double minutes (dmin) is resolved by CGH. However, the drawbacks are that neither balanced translocations, inversions, small deletions nor polyploidization can be detected.

In this study, we compare the copy number changes between mesothelioma and different types of adenocarcinoma and large-cell anaplastic carcinoma (referred to as lung carcinoma in the text). We also evaluate the possibility of using CGH as a tool for distinguishing these two types of tumour. Squamous cell carcinoma of the lung does not usually present a problem for differential diagnosis and was not included. Further analysis using the Southern blot technique was performed using a probe for the *KRAS2* gene to investigate the tumours that showed gains of genetic material in chromosome 12p using CGH.

### MATERIALS AND METHODS

### Mesothelioma

Thirty-four malignant mesotheliomas from patients treated at the Helsinki University Central Hospital were included in the study. Only tumours with a confirmed diagnosis and specimens with sufficient material for successful DNA extraction and CGH analysis were selected for the study. The diagnosis was confirmed by the Finnish National Mesothelioma Panel or by the European Organization for Research and Treatment of Cancer Mesothelioma Panel. There were five fibromatous, 19 epithelial and ten mixed mesotheliomas. Thirty-three of the mesotheliomas were of pleural and one of peritoneal origin. Twenty-four patients had a history of asbestos exposure, nine patients were not aware of any exposure to asbestos, and the asbestos exposure in one patient was not known. Thirty of the specimens were formalin fixed and paraffin embedded; four were fresh frozen tumours (case nos 20, 21, 23 and 24) (Table 1).

### Lung carcinoma

Ten adenocarcinomas, ten bronchoalveolar and ten large-cell anaplastic carcinomas were selected from the files of the Department of Pathology, University of Helsinki (Table 1). The specimens were formalin fixed and paraffin embedded. We selected the ten most recently diagnosed carcinomas in each group with sufficient material for the analyses.

### DNA extraction

Sections were examined and the tumour area was marked. All irrelevant material was cut away and a new paraffin block was made of the remaining tumour tissue that contained at least 60% malignant cells. Thirty 3- to 5 μm-thick sections were cut and DNA extraction was performed as described elsewhere (Miller et al, 1988; Isola et al, 1994). DNA in peripheral blood specimens from healthy donors (male and female) was extracted according to standard procedures and used as reference in the CGH analyses.

### CGH analysis

The CGH analyses were performed according to the method of Kallioniemi et al (1994), with some minor modifications. In brief, 800 ng of fluorescein isothiocyanate (FITC)-dUTP (Du Pont NEN Products, Boston, MA, USA)-labelled tumour DNA and 800 ng of Texas Red-dUTP (Du Pont)-labelled normal reference DNA together with 20 μg of unlabelled human Cot-1 DNA (Gibco BRL, Gaithersburg, MD, USA) in 10 μl of hybridization buffer [50% formamide, 10% dextran sulphate, 2×SSC (1×SSC is 0.15 M sodium chloride/0.015 M sodium citrate, pH 7)] were denatured at 75°C for 5 min and applied to normal lymphocyte metaphase preparations. Before hybridization, the preparations were stored in a fixative solution (methanol–acetic acid, 3:1) for one night, pretreated in 2×SSC at 40°C for 30 min and dehydrated in a series of 70%, 85% and 100% ethanol. The preparations were denatured at 65–67°C for 2 min in a formamide solution (70% formamide/2×SSC) followed by dehydration on ice as described above and treatment with proteinase K. Hybridization (2–3 days at 37°C) was followed by washes to remove unspecifically bound DNA, after which the preparations were counterstained with 4′, 6-diamidino-2-phenyl-indole-dihydrochloride (DAPI; Sigma, St Louis, MO, USA) and covered with an antifade solution (Vector Laboratories, Burlingame, CA, USA). To confirm the CGH results, additional hybridization experiments using the reverse-labelling system, i.e. tumour DNA labelled with Texas Red and reference DNA with FITC, were performed on some specimens.

### Digital image analysis, interpretation and quality control of the CGH results

An Olympus fluorescence microscope and the *isis* digital image analysis system (MetaSystems, Altlussheim, Germany) based on a high-sensitivity integrated monochrome CCD camera and an automated CGH analysis software package were used to analyse the hybridization (for details, see Kivipensas et al, 1996). A region in a chromosome was considered as being over-represented (gained) when the ratio exceeded 1.17 and under-represented (lost) when the ratio was less than 0.85. These cut-off values were based on negative control hybridization experiments, i.e. hybridization of two normal DNAs. Only ratio changes that exceeded the fluctuation seen in the negative control experiments were interpreted as evidence of a real gain or loss of DNA sequences. Furthermore, positive control experiments with tumour DNA of known DNA copy number changes (both losses and gains) were performed to confirm the cut-off values mentioned above. In order to distinguish between different levels of gain/ratios exceeding the values of 1.3 or 1.5 were considered as amplifications or high-level amplifications respectively. Furthermore, intra-experiment standard deviations for every position in the CGH ratio profiles were calculated from the variation of the ratio values of all homologous chromosomes within the experiment. Confidence intervals for the ratio profiles were then calculated by combining them with an empirical inter-experiment standard deviation and estimating error probability of 1% based on the t-distribution. The heterochromatic regions in chromosomes 1, 9 and 16, the p-arms of the acrocentric chromosomes and the Y chromosome were excluded from the analyses because of suppression of hybridization with Cot-1 DNA in these regions. Gains (≥1.17 and <1.3) of genetic material in chromosomes 1p32–pter, 16p, 19 and 22 were not included because of the false-positive results revealed in these chromosomal areas in the negative control experiments.

### Southern blot analysis

The Southern blot method was used to investigate possible amplification of the *KRAS2* gene (probe p640, provided by R Weinberg) in

© Cancer Research Campaign 1998

**Table 1** CGH findings from 34 patients with malignant mesothelioma and 30 patients with primary adeno- or large-cell anaplastic carcinoma

| Case (sex/age at diagnosis/ exposure to asbestos[a]) | Losses (≤ 0.85) | Gains (≥ 1.17 and < 1.3) | Amplifications (≥ 1.3 and < 1.5) | High-level amplifications (≥ 1.5) |
|---|---|---|---|---|
| *Mesothelioma* | | | | |
| Fibromatous | | | | |
| 1 (M/77/?) | 4q31.3–qter | 1q, 6q21–p22, 7, 8 | | |
| 2 (M/63/–) | 4qcen–q26 | 5q23–qter, 14q24–qter | | |
| 3 (M/56/–) | | 5p | | |
| 4 (M/41/+) | 6qcen–q22 | 6p, 15q15–q21 | 15q21–qter | |
| 5 (F/73/–) | | 8p | 7 | |
| Epithelial | | | | |
| 6 (F/69/–) | 6q22–qter, 8p, 10qcen–q23, 17q21–pter | 6q21–pter, 15q, 17q21–qter | 3p22–pter, 3p14–qter, 5 | |
| 7 (M/55/+) | None | None | None | None |
| 8 (M/47/+) | None | None | None | None |
| 9 (M/55/+) | 4, 5q, 9q, 14q, 22q | 5p, 9p | | |
| 10 (M/78/+) | None | None | None | None |
| 11 (M/63/+) | 5q13–q22, 7q31–qter, 13q21–qter, 14q13–qter | 7p | | |
| 12 (M/44/+) | 1pcen–p22, 3p24–pter, 6qcen–q22, 9p, 13qcen–q22, 14q13–q21 | 1q, 2qcen–q14.1, 5q31–qter, 6p, 7q, 8p12–qter, 9q, 11, 12q22–qter | 15q22–qter | |
| 13 (M/61/–) | None | None | None | None |
| 14 (M/79/+) | | 7 | | |
| 15 (M/57/+) | None | None | None | None |
| 16 (M/40/+) | 3qcen–q25, 6, 9p21–pter, 13q13–qter | 2, 7p, 15q21–qter, 21q | | |
| 17 (M/59/+) | 4, 9pter–q22, 10q, 13q, 14q21–qter, 15qcen–q15 | 7q, 11 | 8, 10p | |
| 18 (F/62/–) | 6q | 6p, 9q31–qter, 15q | | |
| 19 (M/53/+) | 6qcen–q21 | 2q24–qter, 11q14–q22 | | |
| 20 (M/66/+) | | 5, 7, 8 | | |
| 21 (M/44/+) | 2q33–qter, 6q22–qter, 12p12–pter | 1qcen–q41, 15qcen–q14, 15q22–qter, 17q21–qter | | |
| 22 (M/53/+) | 1p21–p31, 2q34–qter, 9p, 14q | 1q23–q41, 9q, 11p, 15q23–qter | 1qcen–q23, 1q41–qter 11q14–q22 | 11qcen–q14, 11q22–qter |
| 23 (M/68/+) | 4, 14q, 17p, 18p | 3pcen–p14, 3q, 5p, 7p, 8, 13q21–qter | 15q21–qter | |
| 24 (M/42/+) | 1pcen–p22, 22q | 1q | | |
| Mixed | | | | |
| 25 (M/63/+) | 10, 16, 17p, 22q | 3q13.2–q26.3 | | |
| 26 (M/57/+) | 1p21–p31, 4, 6q15–qter, 10, 13q, 14q13–q23 | 9p | | |
| 27 (M/60/–) | | 5p | | |
| 28 (M/55/+) | 4q24–pter, 9pcen–p22, 11p14–pter, 14q | | | |
| 29 (F/68/–) | Xp, 1p, 3q23–pter, 4q, 5, 6q22–qter, 16q | 4p | | |
| 30 (M/59/+) | 4q33–qter, 16p | 11p | 12p13 | 12pcen–p12 |
| 31 (M/41/+) | None | None | None | None |
| 32 (M/56/+) | 4p15.3–qter, 6q16–qter, 9p, 10q23–pter 14q, 16q | 3p21–pter | 12q22–qter | |
| 33 (M/70/–) | None | None | None | None |
| 34 (M/57/+) | 13q | | | |
| *Lung carcinoma* | | | | |
| Adenocarcinoma | | | | |
| 35 (F/76) | | 1q, 5, 6p, 7p, 8q21.1–q21.2, 8q23–qter, 14q, 17q24–qter | X, 2p13–p16, 8q21.3–q23 | 12q14–q21, 21q |
| 36 (F/79) | 17p | Xq23–qter, 1, 2q22–q24, 3, 5q23–q33, 6q21–qter, 7q, 14q | 5p, 5qcen–q23, 7p, 8q22–qter | |
| 37 (M/80) | 8p, 13q | X, 1, 5q, 10p, 14q22–qter | 14qcen–q21, 8q21.1, 8q24.1–qter | 5p, 7p, 8q21.1–q24.1 |
| 38 (M/63) | | 1q, 6p, 18q | | |
| 39 (M/70) | None | None | None | None |
| 40 (M/48) | 8p | 1, 2p15–q22, 7p, 8q21.3–qter 10qcen–q22, 11qcen–q13 | 2q22–q32, 5p, 8qcen–q21.2, 11q14–qter, 12pcen–p12 | |
| 41 (F/67) | | 8q, 10q | | |
| 42 (M/68) | None | None | None | None |
| 43 (M/52) | 3p, 9p21–pter | Xp21–qter, 1qcen–q32, 5p, 6q23–qter, 12q | 8q23–qter, 12pcen–p13 | |
| 44 (F/46) | 4q24–qter | 1q, 2p23–pter, 6p | | |
| Bronchoalveolar | | | | |
| 45 (M/68) | 4 | 1qcen–q41 | 8pcen–p21 | |
| 46 (M/73) | 6q, 8p, 18 | 5, 6p, 8q | 1q | |
| 47 (M/63) | 6qcen–q23 | 1q, 6p | | |

*© Cancer Research Campaign 1998*

| Case (sex/age at diagnosis/ exposure to asbestos[a]) | Losses ($\leq$ 0.85) | Gains ($\geq$ 1.17 and < 1.3) | Amplifications ($\geq$ 1.3 and < 1.5) | High-level amplifications ($\geq$ 1.5) |
|---|---|---|---|---|
| 48 (F/60) | | 7p21–q36, 8q | | |
| 49 (M/50) | 1p, 17p, 18 | 1q, 2p24–q34, 10p, 13q | 10q | |
| 50 (F/52) | 8p, 12p12–pter, 18q | 8q | | |
| 51 (M/80) | None | None | None | None |
| 52 (M/65) | 6q, 11q23–qter | 2, 6p, 7, 8qcen–q21.1, 10, 11pcen–p15 | 1q, 8q23–qter | 12pcen–p13 |
| 53 (F/56) | None | None | None | None |
| 54 (F/80) | | 1qcen–q41, 7p | | |
| Large-cell anaplastic carcinoma | | | | |
| 55 (M/60) | 10p13–pter | 5p14–pter, 7qcen–q31, 11q, 12q14–qter | 1q22–q31, 7q31–qter, 8q13–q23, 10q24–qter, 18 | 8p12–q12, 8q24.1–qter |
| 56 (M/65) | | 3qcen–q24, 3q27–qter, 5pcen–p15.1, 14qcen–q13, 14q24–qter, 15q22–qter | 3q25–q26 | |
| 57 (M/49) | | 8q21.3–qter | 7q22–q31 | |
| 58 (F/59) | X | 6pcen–p21.3, 7q11.2–pter, 10p, 12p | 3q21–q26.1, 8q | |
| 59 (M/54) | 17p | X, 2pcen–p15, 5q, 8q, 11qcen–q14, 12q13–qter, 13q, 18qcen–q21 | 1q, 5p, 11pcen–p14 | |
| 60 (M/60) | 9p21–pter | 1q, 6p, 8q22–qter | 1p31–p36.1, 12pcen–p13 | |
| 61 (M/57) | | 7 | | |
| 62 (F/72) | X, 6qcen–q23 | 2q32–qter, 12 | 18 | |
| 63 (M/65) | 13q21–q32 | 8qcen–q22 | 7q11.2–pter, 8q23–qter | |
| 64 (F/45) | X | 2pter–q14.2, 10, 11qcen–q21, 12 | 4p, 7p, 8p12–qter | |

[a]Mesothelioma patients. ?, Asbestos exposure not known; +, asbestos exposure; –, no asbestos exposure.

**Table 2** Discriminant analysis of histological diagnosis and CGH findings. Method of prediction: (A) linear discriminant analysis; (B) quadratic discriminant analysis

**A**

| Histological diagnosis | Lung carcinoma | | | Mesothelioma | | |
|---|---|---|---|---|---|---|
| | Adenocarcinoma | Bronchoalveolar | Large-cell anaplastic | Epithelial | Fibromatous | Mixed |
| Adenocarcinoma | 4 | – | 3 | – | 1 | – |
| Bronchoalveolar | – | 2 | 2 | 3 | – | 1 |
| Large-cell anaplastic | 3 | – | 5 | 2 | – | – |
| Epithelial | – | – | 2 | 11 | – | 1 |
| Fibromatous | – | 1 | 2 | 2 | – | – |
| Mixed | – | 1 | – | 1 | – | 6 |

**B**

| Histological diagnosis | Lung carcinoma | Mesothelioma |
|---|---|---|
| Lung carcinoma | 17 | 9 |
| Mesothelioma | 3 | 24 |

the tumours that had gains, amplifications or high-level amplifications in the short arm of chromosome 12 in the CGH analyses. DNA was available in seven of the eight tumours with gain in 12p (case nos 30, 40, 43, 52, 58, 60 and 62). Case no. 38, for which CGH revealed a normal chromosome 12p, and a normal blood sample were used as negative controls. The p105–153A probe hybridizing to chromosome 5q11.2–13.3 was chosen as a control probe because of normal CGH results in this region in the tumours tested for *KRAS2* amplification. *Hin*dIII-digested DNA samples were hybridized with p640 and rehybridized with reference probe p105–153A. Probes p640 and p105–153A hybridize to fragments of approximately 1 kb and 3 kb respectively. The analysis and interpretation of the results were performed as described elsewhere (Peltomäki et al, 1991; Monni et al, 1996).

### Multivariate analysis

The calculated frequencies of DNA copy number changes and the statistical analyses were based on those tumours that had either gains or losses of genetic material.

© Cancer Research Campaign 1998 

**Table 3** Descriptive statistics of the frequency of chromosomal gains and losses in malignant mesothelioma and adeno- or large-cell anaplastic carcinoma

| Type | Mean | Standard deviation | Minimum | Maximum |
|---|---|---|---|---|
| *Gains* | | | | |
| Lung carcinoma | 6.0 | 4.1 | 1 | 13 |
| Adenocarcinoma | 7.8 | 4.5 | 2 | 13 |
| Bronchoalveolar | 4.0 | 3.3 | 1 | 11 |
| Large-cell anaplastic | 6.3 | 4.1 | 2 | 13 |
| Mesothelioma | 3.2 | 2.9 | – | 12 |
| Mixed | 1.0 | 0.8 | – | 2 |
| Epithelial | 4.6 | 3.2 | 1 | 12 |
| Fibromatous | 3.0 | 2.3 | 1 | 7 |
| *Losses* | | | | |
| Lung carcinoma | 1.3 | 1.2 | – | 4 |
| Adenocarcinoma | 0.9 | 0.8 | – | 2 |
| Bronchoalveolar | 2.0 | 1.6 | – | 4 |
| Large-cell anaplastic | 1.1 | 1.0 | – | 3 |
| Mesothelioma | 3.4 | 2.9 | – | 9 |
| Mixed | 4.9 | 3.5 | – | 9 |
| Epithelial | 3.5 | 2.3 | – | 7 |
| Fibromatous | 0.6 | 5.5 | – | 1 |

**Table 4** Statistically significant differences between mesothelioma and lung carcinoma

| DNA copy number changes | Mesothelioma (%) | Lung carcinoma (%) | RR | 95% CI |
|---|---|---|---|---|
| *Gains* | | | | |
| Xp | – | 15 | 0 | 0–0.88 |
| Xq | – | 19 | 0 | 0–0.69 |
| 1p | – | 15 | 0 | 0–0.87 |
| 1q | 19 | 62 | 0.30 | 0.13–0.66 |
| 2p | 4 | 26 | 0.14 | 0.023–0.78 |
| 8q | 19 | 65 | 0.28 | 0.12–0.61 |
| 10q | – | 23 | 0 | 0–0.57 |
| 12p | 4 | 27 | 0.14 | 0.020–0.78 |
| 15q | 30 | 4 | 7.7 | 1.4–46.7 |
| 18q | – | 15 | 0 | 0–0.88 |
| *Losses* | | | | |
| 4p | 22 | 4 | 5.8 | 1.0–35.9 |
| 4q | 37 | 8 | 4.8 | 1.3–18.7 |
| 10q | 19 | – | [a] | 1.34–[a] |
| 14q | 33 | – | [a] | 2.5–[a] |

[a]Category not applicable. RR, risk ratio; CI, confidence interval.

Discriminant analysis was used to distinguish between diagnostic groups based on observed DNA copy number changes. We began by using a linear discriminant function (Fisher, 1936) as the statistical criterion for classification of the tumours into six separate diagnostic groups. The first discriminant function (or canonical variate) was taken as the linear combination of the frequency of the total number of losses of DNA sequences and the total number of gains separately in the p-arm and in the q-arm; the components were coded as four predictor variates (Gp, Gq, Lp, Lq). These discrete variates were subjected to the Freeman–Tukey transformation (i.e. $\sqrt{Gp} + \sqrt{Gp + 1}$) to approximate the normal distribution (see Johnson and Kotz, 1969, p. 99). The linear discriminant function has a maximal ratio of the separation of the group means to the within-group variance. The second discriminant function is the linear combination that is uncorrelated (but not necessarily orthogonal) to the first, which has the same optimality criterion. The third discriminant function is defined analogously. A tumour was classified by calculating its Euclidean distance from the diagnostic group centroids, projected onto a subspace defined by a subset of the canonical variates. The tumour was assigned to the closest group. The program output contained a discriminant function score for each tumour and group mean values. We also applied quadratic discrimination to these data. The alternative allocation rule uses the smallest expected number of errors as the

 © Cancer Research Campaign 1998

**Figure 1** Gains and losses of DNA sequences in 27 mesotheliomas and 26 lung carcinomas. Losses are shown on the left side of the chromosome and gains on the right. The first chromosome in a pair represents mesothelioma (MM), the second one represents lung carcinoma (LC). Dotted lines are amplifications (gains ≥ 1.3 and < 1.5); bold lines are high-level amplifications (gains ≥ 1.5). Only chromosomes with changes are shown selection criterion for allocating a tumour to the α priori specified diagnostic group to which it has the maximum a posteriori probability of belonging. This Bayes rule can be linked to the Mahalanobis distance (Mahalanobis, 1936) by assuming that the groups are jointly distributed multivariate normal within the same covariance matrix. To calculate the above discriminant analysis functions, we used the *discr*, *ida* and *qda* programs implemented in the S-PLUS system (Venables and Ripley, 1994).

### Univariate analysis

The preceding multivariate analysis was supplemented with univariate analyses of changes in a specific chromosome. This strategy was adopted because the multivariate method discriminated between groups based on histological diagnosis, and of interest was a comparison of different combinations of subgroups formed on the basis of morphological characteristics. A comparison of the relative frequency of the occurrence of DNA copy number changes in a single chromosome between malignant mesothelioma and lung carcinomas was carried out in terms of the risk ratio (RR) parameter using the method of Miettinen and Nurminen (1985).

### RESULTS

### Comparison of the CGH results for mesothelioma and lung carcinoma

#### *Multivariate analysis*

Table 2A gives the cross-classification of the 53 informative tumours into six separate subgroups based, on one hand, on the histological diagnosis and, on the other hand, on the predicted diagnosis by the linear discriminant function analysis of chromosomal changes (gains and losses in the p- and q-arm). The overall misclassification rate was 47%. When focusing on the mesothelioma–lung carcinoma discrimination, 3 (case nos 1, 20 and 23) of 27 mesotheliomas and 9 (case nos 44–47, 50, 54, 61–63) of 26 lung carcinomas were incorrectly classified by the quadratic discriminant analysis (Table 2B). Thus the sensitivity of CGH to differentiate a mesothelioma from a lung carcinoma was 89% and

© Cancer Research Campaign 1998

**Figure 2** Selected CGH profiles of the most frequent gains and losses of DNA sequences in mesothelioma and lung carcinoma. The chromosome numbers are shown under the profiles and the case numbers on top. The line in the middle is the base line ratio (1.0); the left and the right lines indicate ratio values of 0.85 and 1.17

**Figure 3** Southern blot analysis of tumours with gains of genetic material in 12p. Lane 1 represents case no. 43 and lane 2 case no. 40. Lanes 3 and 4 represent negative controls: case no. 38 and a normal blood sample respectively. CGH profiles of chromosome 12 in case nos 38, 40 and 43 are also shown the specificity 63%. When the gains and losses in both arms were combined, the overall error rate was 21% with 81% sensitivity and 77% specificity.

### *Univariate analysis*

Table 3 gives the mean value and standard deviation of the number of gains and losses of DNA sequences detected in the two different main types of tumour and in the separate histological subgroups. Although differences in the frequency of gains and losses were detected between mesothelioma and lung carcinoma, they were not statistically significant (Fisher's exact test). However, when focusing on separate chromosomes, significant differences were seen in X, 1, 2p, 4, 8q, 10q, 12p, 14q, 15q and 18q (Table 4).

There was no statistically significant difference between the DNA copy number changes detected in separate chromosomes in the three histological subgroups of mesothelioma. When combining tumours from the fibromatous and mixed group, which is permissible because of clinical and prognostic similarities, a gain of genetic material in 15q was found to be more common in epithelial tumours ($n = 14$) than in the fibromatous mixed group ($n = 13$) [risk ratio (RR) 6.5, 95% confidence interval (CI) 1.3–39.3].

Statistically significant differences in losses and gains of genetic material were not detected between the three types of lung carcinoma. When considering adenocarcinoma and bronchoalveolar tumours as one group ($n = 16$), a gain in 1q occurred more often in them than in the tumours in the large-cell anaplastic carcinoma group ($n = 10$) (RR 2.7, 95% CI 1.2–7.8).

### Mesothelioma

Twenty-seven out of the 34 mesotheliomas showed DNA copy number changes. Gains of genetic material occurred as frequently as losses (Table 3). High-level amplifications were only detected in 11q and 12p (Table 1 and Figure 1).

The most common aberration in the mesotheliomas was a loss of DNA sequences in the long arm of chromosomes 4 and 6 in 10 of the 27 (37%) informative tumours. The minimal common region of loss extended in chromosome 4 from the 4q centromere to band q24 and 4q33 to the q-telomere and in chromosome 6 it was only band q22. Losses occurred frequently in the long arms of chromosomes 13 (q21–q22) and 14 (q21) and in the short arm of chromosome 9 (p21) in 22%, 33% and 22% of the abnormal specimens respectively (Table 1, Figures 1 and 2).

The most recurrent gain of DNA sequences was detected in the long arm of chromosome 15 (q23–qter) in 9 of the 27 (33%) informative cases. There were three amplifications among these gains. Other regions commonly gained in the abnormal tumours were the short arms of chromosomes 5 (pcen-pter, 22%), 7 (pcen-pter, 26%) and 8 (pcen-p12, 22%) and the long arm of chromosome 7 (qcen–qter, 22%). Among these gains one amplification was detected per chromosome (Table 1, Figures 1 and 2).

### Lung carcinoma

DNA copy number changes were detected in 26 of the 30 specimens evaluated. Gains of genetic material predominated over losses with a ratio of 4.6:1 (Table 3). There were high-level amplifications in 5p, 7p, 8p, 8q, 12p, 12q and 21q (Table 1, Figures 1 and 2).

A gain in DNA sequences in the long arm of chromosome 8 (q23–qter) was the most recurrent aberration found in 17 of the 26 (65%) informative tumours. Seven of these were amplifications and three were high-level amplifications. More than half (62%) of the informative specimens had gains in the long arm of chromosome 1 (q22–q31). Four amplifications and 12 gains were observed in this area. Gains were also frequent in the short arms of chromosomes 6 (pcen–p21.3, 31%), 5 (p14, 35%) and 7 (pcen–p21, 42%). The last two included one high-level amplification and three amplifications (Table 1, Figures 1 and 2).

Losses of DNA sequences were most common in the long arm of chromosome 6 (qcen–q23). These aberrations were found in 4 of the 26 (15%) abnormal tumours. Other chromosomal areas that were lost in three or four tumours were the short arms of chromosomes 8 (pcen–pter, 15%) and 17 (pcen–pter, 12%), the long arm of chromosome 18 (qcen–qter; 12%) and the whole X chromosome (pter–qter; 12%) (Table 1, Figures 1 and 2).

© Cancer Research Campaign 1998

### Southern blot analysis

Amplification of *KRAS2* was detected in two of the seven tumours with a gain of genetic material in 12p. Both of these were regular adenocarcinomas (case nos 40 and 43). Compared with the negative controls, these carcinomas showed increased dosages (3.7- and 8.2-fold) of *KRAS2* (Figure 3). In the other five tumours, the analysis failed because of the poor quality of the DNA.

### DISCUSSION

The main result in our study is that there is a difference between the pattern of DNA copy number changes in mesothelioma and that in lung carcinoma. By combining the occurrence of gains and losses of genetic material in the individual tumours, we were able to predict the correct type of tumour in 41 of the 53 informative cases. When comparing DNA copy number changes in single chromosomes, significant differences were detected in ten chromosomes.

Discriminant analysis for normal populations assumes that the joint distribution of all predictors is multivariate normal. In practice, this assumption is not always valid, and even the predictor variates are only approximately normal. Therefore we have to rely on the robustness of the applied procedure to depart from normality. The detected chromosome changes were originally coded as 63 indicator variates. The sum of binary (0, 1) variates tends to be normally distributed, and the transformation of the summed variates helps to approximate this distributional assumption. (We note parenthetically that the reduction of the number of variates must be performed without regard to their relationship to the outcome variate, i.e. the type of tumour – otherwise the selection procedure will be biased.) To check the stability of the results, we conducted a logistic discriminant analysis that makes fewer assumptions about the distributions of the variates. This method yielded results similar to those obtained by the *ida* and *qda* methods.

Nevertheless, the size of the subgroups of classified tumours was too small – in particular, there were only five fibromatous mesotheliomas – to form reliable predictor models. In practice, in order to have predictive discrimination that validates a new series of tumours, the number of variates selected for the discriminant function should be no more than the number of tumours in the sample that was used in fitting the model divided by ten (Harrel et al, 1996). The size rule applies because we used four (or two) summary variates on a sample of 53 (i.e. $4 < 53/10 \sim 5$). To discriminate between the two diagnoses (mesothelioma and lung carcinoma), the number of tumours in the less frequent group (26) should be at least roughly ten times higher than the number of predictors ($10 \times 4 = 40$ or $10 \times 2 = 20$); here the rule does not apply when four predictors are used.

Our primary measure of accuracy of classification was the error (or misclassification) rate, as this is the quantity that the Bayes rule minimizes. The most stringent test of a predictor model is an external validation – the application of the estimated model to a new patient population. Unfortunately, we did not have another series of tumours to test the performance of the model. However, the error rate on a randomly chosen set from the whole population will be an unbiased estimator. For this cross-validation (Efron, 1983), we first randomly allocated 53 tumours into ten mutually exclusive subsamples. We then left out a subsample, estimated the discriminant function model on the remaining sample and used the fitted model to classify the previously drawn subsample. We replicated this procedure for the other nine subsamples. The cross-validated error rate was then formed by averaging one minus the posterior probability assigned to the selected class. Another advantage of this technique is that it does not depend on the correctness of the supplied classification based on the histological diagnosis (Venables and Ripley, 1994). The cross-validated result for the previously obtained error rate of 21% was 26%, indicating a fair reliability of the model to discriminate between mesothelioma and lung carcinoma.

The DNA copy number changes detected in mesothelioma in this analysis, such as losses of genetic material in 1p, 4q, 6q, 9p, 13q, 14q and gains in 5p and 7p, are supported by previous cytogenetic and CGH studies, although with some differences in frequency of occurrence (Tiainen et al, 1989; Hagemeijer et al, 1990; Taguchi et al, 1993; Kivipensas et al, 1996; Björkqvist et al, 1997). These chromosomal regions probably carry important genes for the development and progression of mesothelioma. Losses of DNA sequences in 13q were detected in six tumours in this study. Five of these showed a loss in 13q14 in which the *RB1* tumour-suppressor gene is located. However, a study by Van der Meeren et al (1993) on mesothelioma cell lines suggests that inactivation of *RB1* is not a critical step in the development of mesothelioma.

The most common copy number changes in non-small-cell lung carcinoma (NSCLC) in this study were gains in 8q, 1q, 7p, 5p and 6p (in decreasing order of frequency). Cytogenetic analyses of NSCLC have detected, on average, more losses than gains of chromosomal material (Lukeis et al, 1990; Testa et al, 1994). These results are to some extent in contrast to ours, because we detected over four times more gains than losses. However, gains in 1q, 7 and 12q have been frequent findings by cytogenetic analysis (Lukeis et al, 1990; Testa et al, 1994) and they were also frequent in our study. Because marker chromosomes are common cytogenetic findings, it is obvious that some of the chromosomal material thought to be lost resides in them. CGH is a DNA-based method and therefore the genetic material in marker chromosomes as well as in dmin is also analysed. Furthermore, CGH reveals only clonal aberrations that exist in at least 50% of the cells, meaning that clonal aberrations found only in a small proportion of the cells will not be detected (Kallioniemi et al, 1994). Some of the DNA copy number changes seen in our study, particularly high-level amplifications, represent new findings that may have an important role in the tumorigenesis of NSCLC. Gains of genetic material in the long arm of chromosome 8 are not often found in cytogenetic analyses. However, the presence of isochromosome 8q has been associated with primary adenocarcinomas (Jin et al, 1988) and gains in 8q have been reported to be frequent in pleural effusions from NSCLC patients (Lukeis et al, 1993). In our CGH analysis, this particular aberration (including three high-level amplifications) occurred in 65% of the informative tumours. Amplification of the *MYC* oncogene has been detected in some NSCLCs (Cline and Battifora, 1987; Slebos et al, 1989). *MYC* resides in the minimal common region of overlap (8q23–qter) in our study and therefore it is likely to be one of the amplified genes.

The difference in the occurrence of losses and gains of genetic material detected in our study may suggest that mesotheliomas and lung carcinomas develop and progress in different ways. This hypothesis is supported by molecular analyses that have demonstrated that mutations in the tumour-suppressor gene *P53* in 17p and the oncogene *KRAS2* in 12p are frequent in NSCLCs but not in mesotheliomas (Metcalf et al, 1992; Ridanpää et al, 1994). We detected amplification of *KRAS2* in two adenocarcinomas and a

© Cancer Research Campaign 1998

gain of genetic material in 12p in seven carcinomas, supporting the role of gene amplification as an alternative pathway by which *KRAS2* is activated.

Similarities, such as gains of genetic material in 5p, 6p and 7p, between mesothelioma and lung carcinoma were also found. We detected a gain in 7p in seven mesotheliomas and 11 lung carcinomas (including one high-level amplification). The *EGFR* gene, located in 7p12–p13, may be one of the altered genes and may therefore be important in the tumorigenesis of both types of tumour. The putative tumour-suppressor genes *MTS2* and *MTS1* in 9p21 are deleted or mutated in both types of tumour (Xiao et al, 1995*a* and *b*). We detected deletions in 9p in six mesotheliomas but only in two carcinomas. Based on previous published cytogenetic data on mesothelioma and NSCLC, a higher frequency of losses in 9p was to be expected (Hagemeijer et al, 1990; Lukeis et al, 1990; Taguchi et al, 1993; Testa et al, 1994). It is likely that deletions in 9p existed in our specimens but were not detected because of intratumour genetic heterogeneity.

In conclusion, we found differences in DNA copy number changes between mesothelioma and lung carcinoma, suggesting that they are genetically different tumour entities. Although CGH cannot be used as a definitive discriminatory method, based on the CGH results, we were able to distinguish between mesothelioma and lung carcinoma in 77% of the abnormal cases. In addition, our CGH results of primary adenocarcinoma and large-cell anaplastic carcinoma of the lung revealed new findings of losses, gains and amplifications of genetic material, which could be important for their development and progression.

### ACKNOWLEDGEMENTS

The authors thank Terhi Kiviranta, Tuula Niilola, Aino Kyyhkynen and Leena Luostarinen for technical assistance. This work was supported by grants from the Finnish Cancer Society.

### REFERENCES

Björkqvist A-M, Tammilehto L, Anttila S, Mattson K and Knuutila S (1997) Recurrent DNA copy number changes in 1q, 4q, 6q, 9p, 13q, 14q and 22q detected by comparative genomic hybridization in malignant mesothelioma. *Br J Cancer* **75**: 523–527

Brown RW, Clark GM, Tandon AK and Allred DC (1993) Multiple-marker immunohistochemical phenotypes distinguishing malignant pleural mesothelioma from pulmonary adenocarcinoma. *Hum Pathol* **24**: 347–354

Chahinian AP, Pajak TF, Holland JF, Norton L, Ambinder RM and Mandel EM (1982) Diffuse malignant mesothelioma. Prospective evaluation of 69 patients. *Ann Internal Med* **96**: 746–755

Cline MJ and Battifora H (1987) Abnormalities of protoonocogenes in non-small cell lung cancer, correlations with tumor type and clinical characteristics. *Cancer* **60**: 2669–2674

Doglioni C, Dei Tos AP, Laurino L, Iuzzolini P, Chiarelli C, Celio MR and Viale G (1996) Calretinin: a novel immunocytochemical marker for mesothelioma. *Am J Surg Pathol* **20**: 1037–1046

Edwards C and Oates J (1995) OV 632 and MOC 31 in the diagnosis of mesothelioma and adenocarcinoma: an assessment of their use in formalin fixed and paraffin wax embedded material. *J Clin Pathol* **48**: 626–630

Efron B (1983) Estimating the error rate of a prediction rule: improvement on cross-validation. *J Am Stat Assoc* **78**: 36–48

Fisher RA (1936) The use of multiple measurements in taxonomic problems. *Ann Eug* **7**: 179–188

Hagemeijer A, Versnel MA, Van Drunen E, Moret M, Bouts MJ, van der Kwast TH and Hoogsteden HC (1990) Cytogenetic analysis of mesothelioma. *Cancer Genet Cytogenet* 1–28

Harrel F, Lee K and Mark K (1996) Multivariate prognostic models: issues in developing models, evaluating assumptions and adequacy, and measuring and reducing errors. *Stat Med* **15**: 361–387

Hirvonen A, Saarikoski ST, Linnainmaa K, Koskinen K, Husgafvel-Pursiainen K, Mattson K and Vainio H (1996) Glutathione S-transferase and N-acetyltransferase genotypes and asbestos-associated pulmonary disorders. *J Natl Cancer Inst* **88**: 1853–1856

Isola J, DeVries S, Chu L, Ghazvini S and Waldman F (1994) Analysis of changes in DNA sequence copy number by comparative genomic hybridization in archival paraffin-embedded tumor samples. *Am J Pathol* **145**: 1301–1308

Jin Y-s, Mandahl N, Heim S, Schuller H and Mitelman F (1988) Isochromosomes i(8q) or i(9q) in three adenocarcinomas of the lung. *Cancer Genet Cytogenet* **33**: 11–17

Johnson NL and Kotz S (1969) *Discrete Distributions*. Wiley: New York

Kallioniemi A, Kallioniemi O-P, Sudar D, Rutovitz D, Gray JW, Waldman F and Pinkel D (1992) Comparative genomic hybridization for molecular cytogenetic analysis of solid tumors. *Science* **258**: 818–821

Kallioniemi O-P, Kallioniemi A, Piper J, Isola J, Waldman FM, Gray JW and Pinkel D (1994) Optimizing comparative genomic hybridization for analysis of DNA sequence copy number changes in solid tumors. *Genes Chromosom Cancer* **10**: 231–243

Kivipensas P, Björkqvist A-M, Karhu R, Pelin K, Linnainmaa K, Tammilehto L, Mattson K, Kallioniemi O-P and Knuutila S (1996) Gains and losses of DNA sequences in malignant mesothelioma by comparative genomic hybridization. *Cancer Genet Cytogenet* **89**: 7–13

Lukeis R, Irving L, Garson M and Hasthorpe S (1990) Cytogenetics of non-small cell lung cancer: analysis of consistent non-random abnormalities. *Genes Chromosom Cancer* **2**: 116–124

Lukeis R, Ball D, Irving L, Garson OM and Hasthorpe S (1993) Chromosome abnormalities in non-small cell lung cancer pleural effusions: cytogenetic indicators of disease subgroups. *Genes Chromosom Cancer* **8**: 262–269

Mahalanobis PC (1936) On the generalized distance in statistics. *Proc Natl Inst Sci India* **12**: 49–55

Metcalf RA, Welsh JA, Bennett WP, Seddon MB, Lehman TA, Pelin K, Linnainmaa K, Tammilehto L, Mattson K, Gerwin Bl and Harris CC (1992) p53 and Kirsten-*ras* mutations in human mesothelioma cell lines. *Cancer Res* **52**: 2610–2615

Miettinen M and Kovatich AJ (1995) HBME-1 a monoclonal antibody useful in the differential diagnosis of mesothelioma, adenocarcinoma, and soft-tissue and bone tumors. *Appl Immunohistochem* **3**: 115–122

Miettinen O and Nurminen M (1985) Comparative analysis of two rates. *Stat Med* **4**: 213–226

Miller SA, Dykes DD and Polesky HF (1988) A simple salting out procedure for extracting DNAs from human nucleated cells. *Nucleic Acids Res* **16**: 1215

Monni O, Joensuu H, Franssila K and Knuutila S (1996) DNA copy number changes in diffuse large B-cell lymphoma – comparative genomic hybridization study. *Blood* **87**: 5269–5278

Peltomäki P, Alfthan O and de la Chapelle A (1991) Oncogenes in human testicular cancer: DNA and RNA studies. *Br J Cancer* **63**: 851–858

Pisani RJ, Colby TV and Williams DE (1988) Malignant mesothelioma of the pleura. *Mayo Clin Proc* **63**: 1234–1244

Ridanpää M, Karjalainen A, Anttila S, Vainio H and Husgafvel-Pursiainen K (1994) Genetic alterations in p53 and K-*ras* in lung cancer in relation to histopathology of the tumor and smoking history of the patient. *Int J Oncol* **5**: 1109–1117

Skov BG, Lauritzen AF, Hirsh FR, Skov T and Nielsen HW (1994) Differentiation of adenocarcinoma of the lung and malignant mesothelioma: predictive value and reproducibility of immunoreactive antibodies. *Histopathology* **25**: 431–437

Slebos RJ, Evers SG, Wagenaar SS and Rodenhuis S (1989) Cellular protooncogenes are infrequently amplified in untreated non-small cell lung cancer. *Br J Cancer* **59**: 76–80

Taguchi T, Jhanwar SC, Siegfried JM, Keller SM and Testa JR (1993) Recurrent deletions of specific chromosomal sites in 1p, 3p, 6q, and 9p in human malignant mesothelioma. *Cancer Res* **53**: 4349–4355

Testa JR, Siegfried JM, Liu Z, Hunt JD, Feder MM, Litwin S, Zhou J-Y, Taguchi T and Keller SM (1994) Cytogenetic analysis of 63 non-small cell lung carcinomas: recurrent chromosome alterations amid frequent and widespread genomic upheaval. *Genes Chromosom Cancer* **11**: 178–194

Tiainen M, Tammilehto L, Rautonen J, Tuomi T, Mattson K and Knuutila S (1989) Chromosomal abnormalities and their correlations with asbestos exposure and survival in patients with mesothelioma. *Br J Cancer* **60**: 618–626

Van der Meeren A, Seddon MB, Kispert J, Harris CC and Gerwin BI (1993) Lack of expression of the retinoblastoma gene is not frequently involved in the genesis of human mesothelioma. *Eur Respir Rev* **3**: 177–179

© Cancer Research Campaign 1998

Venables WN and Ripley BD (1994) *Modern Applied Statistics with S-plus*. Springer: New York

Wagner JC, Sleggs CA and Marchand P (1960) Diffuse pleural mesotheliomas and asbestos exposure in the Northwestern Cape Province. *Br J Ind Med* **17**: 260–271

Weiss LM and Battifora H (1993) The search for the optimal immunohistochemical panel for the diagnosis of malignant mesothelioma. *Hum Pathol* **24**: 345–346

Xiao S, Li D, Corson JM, Vijg J and Fletcher JA (1995*a*) Codeletion of *p15* and *p16* genes in primary non-small cell lung carcinoma. *Cancer Res* **55**: 2968–2971

Xiao S, Li D, Vijg J, Sugarbaker DJ, Corson JM and Fletcher JA (1995*b*) Codeletion of *p15* and *p16* in primary malignant mesothelioma. *Oncogene* **11**: 511–515

© Cancer Research Campaign 1998